# Manipulating Electron Structure through Dual-Interface Engineering of 3C-SiC Photoanode for Enhanced Solar Water Splitting

**DOI:** 10.1021/jacs.5c04005

**Published:** 2025-04-17

**Authors:** Hui Zeng, Satoru Yoshioka, Weimin Wang, Zhongyuan Han, Ivan G. Ivanov, Hongwei Liang, Vanya Darakchieva, Jianwu Sun

**Affiliations:** † Department of Physics, Chemistry and Biology (IFM), Linköping University, Linköping, SE-58183, Sweden; ‡ Department of Applied Quantum Physics and Nuclear Engineering, Kyushu University, Motooka 744, Nishi-ku, Fukuoka 819-0395, Japan; § MAX IV Laboratory, Fotongatan 2, Lund, SE-22484, Sweden; ∥ School of Integrated Circuits, Dalian University of Technology, Dalian 116024, China; ⊥ NanoLund and Solid State Physics, Lund University, S-22100 Lund, Sweden

## Abstract

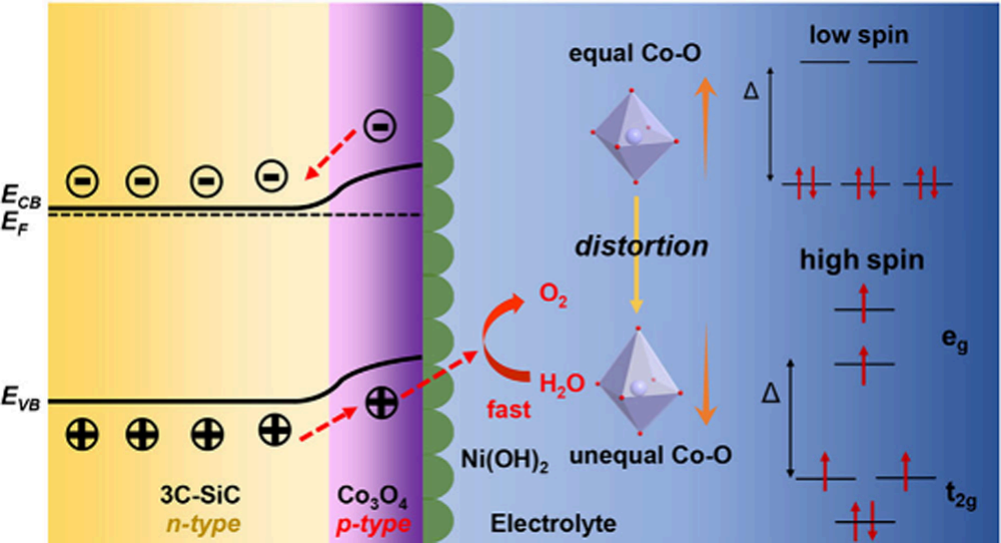

Interface engineering is crucial for enhancing the efficiency of semiconductor-based solar energy devices. In this work, we report a novel dual-interface engineering strategy by designing a Ni(OH)_2_/Co_3_O_4_/3C-SiC photoanode that achieves remarkable enhancements in photoelectrochemical (PEC) water splitting performance. The optimized photoanode delivers a photocurrent density of 1.68 mA cm^–2^ at 1.23 V vs the reversible hydrogen electrode (RHE), representing an 8-fold increase compared to pristine 3C-SiC, along with excellent operational stability. In this architecture, Co_3_O_4_ serves as a highly efficient hole-extraction layer and forms a p–n junction with 3C-SiC, enhancing the separation of photogenerated electron–hole pairs. At the Ni(OH)_2_/Co_3_O_4_ interface, the formation of Ni–O–Co bonds facilitates rapid charge transfer and accelerates oxygen evolution reaction (OER) kinetics. The microwave photoconductivity decay (μ-PCD) measurements confirm a prolonged minority carrier lifetime, demonstrating the critical role of electronic structure modulation in improving charge separation and reducing recombination. Using advanced synchrotron radiation and X-ray absorption spectroscopy, we unveil critical modifications to the interfacial electronic structure induced by the dual-interface engineering and their roles in enhancing PEC performance. These findings establish a clear relationship between electronic structure modulation, charge carrier dynamics, and PEC performance, providing new insights into interface design strategies for highly efficient solar-driven water splitting systems.

## Introduction

Photoelectrochemical (PEC) water splitting offers a sustainable method for converting solar energy into green hydrogen fuel.^1−4^ Cubic silicon carbide (3C-SiC), a third-generation semiconductor, is a promising candidate for this application due to its near-ideal band gap of 2.36 eV.^5^ However, its practical application is hindered by challenges such as bulk and interfacial charge recombination, sluggish water oxidation kinetics, and surface oxidation during PEC reactions.^6,7^ These issues, common to many semiconductor photoelectrodes, significantly limit their solar-to-hydrogen conversion efficiency.

To address these limitations, strategies such as structural tuning, heterojunction formation, and surface decoration with oxygen evolution cocatalysts (OECs) have been employed.^8^ While these approaches can improve PEC performance and mitigate photocorrosion, many OEC-modified photoelectrodes still face low efficiency due to weak interfacial coupling, which hampers effective extraction of photogenerated holes.^9^ Therefore, enhancing the interfacial coupling between photoelectrodes and cocatalysts is crucial for improving charge separation and optimizing PEC water splitting.

Interfacial engineering plays a vital role in improving charge separation and boosting the PEC performance of photoanodes. A critical aspect is the buried interface between the semiconductor and the OEC, which governs hole extraction. Introducing a hole-extraction layer (HEL) at this interface can significantly enhance PEC activity.^10^ In this work, we propose a p-type semiconductor as the interfacial HEL to form a p–n junction with 3C-SiC, providing a strong driving force for efficient hole extraction and charge transfer to the OEC. This design improves band alignment, facilitates charge transfer, and enhances oxygen evolution reaction (OER) efficiency. Previous studies have demonstrated the effectiveness of p-type interfacial layers such as NiO_*x*_, CoO_*x*_, and CuO_*x*_ in achieving these outcomes.^11,12^

We have demonstrated that Co_3_O_4_, when integrated with 3C-SiC, offers favorable band alignment and creates an energy landscape conducive to efficient charge separation and transfer.^13^ Previous studies have shown that Co_3_O_4_ forms p–n junctions in other photoanode systems, such as α-Fe_2_O_3_, where the built-in electric field enhances PEC performance.^14^ However, detailed insights into its electronic structure and chemical bonding at the Co_3_O_4_–OEC interface remain limited. The complex nature of multilayer films presents challenges for accurately identifying the electronic structure of Co_3_O_4_ in these configurations. Understanding Co_3_O_4_–OEC interactions is essential to optimize charge transfer dynamics and improve PEC performance, particularly in systems where electronic structure regulation is crucial to enhancing overall efficiency.

In this work, we developed a novel dual interfacial layer strategy by designing a Ni(OH)_2_/Co_3_O_4_/3C-SiC photoanode that significantly enhances charge transfer and stability. Co_3_O_4_ serves as an efficient hole extractor to transfer photogenerated holes from 3C-SiC to the Ni(OH)_2_ cocatalyst, thus minimizing electron–hole recombination. The formation of Ni–O–Co bonds at the interface provides robust charge transfer pathways, leading to significant improvements in PEC performance. This innovative design achieved a highly stable photoanode with a photocurrent density of 1.68 mA cm^–2^ at 1.23 V vs the reversible hydrogen electrode (RHE), which is nearly eight times higher than pristine 3C-SiC. Through advanced structural characterizations, including X-ray photoelectron spectroscopy (XPS), X-ray absorption spectroscopy (XAS), and microwave photoconductivity decay (μ-PCD) measurements, we identified strong interfacial electronic coupling between the interfacial layer and confirmed significantly prolonged minority carrier lifetimes, demonstrating the critical role of dual-interface engineering in achieving enhanced charge transfer. These findings highlight the synergistic relationship between electronic structure modulation, charge carrier dynamics, and PEC performance, underscoring the importance of interface design strategies in developing highly efficient and long-term stable PEC water splitting systems.

## Results and Discussion

**Figure 1:**
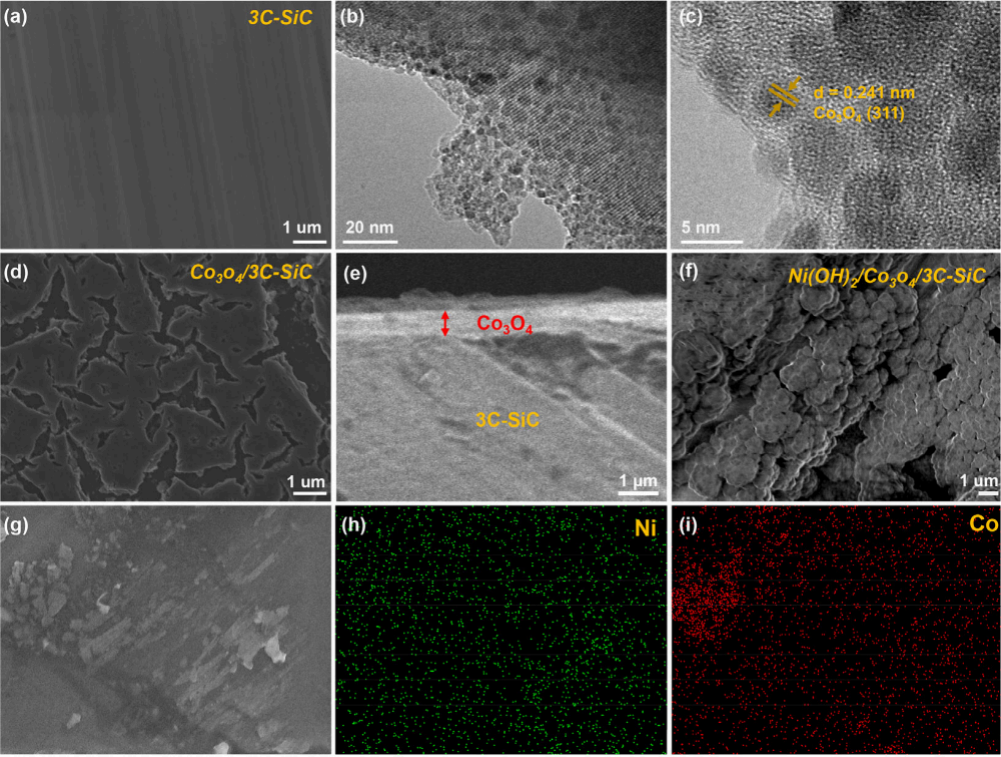
(a) Top view SEM images of 3C-SiC, (b) TEM image of Co_3_O_4_ colloidal nanoparticles, (c) HRTEM of Co_3_O_4_ colloidal nanoparticles, (d) Top view SEM images of Co_3_O_4_/3C-SiC, (e) Cross-sectional SEM image of Co_3_O_4_/3C-SiC, (f) Top view SEM image of Ni(OH)_2_/Co_3_O_4_/3C-SiC, (g) EDS elemental mapping of the Ni(OH)_2_/Co_3_O_4_/3C-SiC. Elemental distribution of (h) Ni and (i) Co.

The Ni(OH)_2_/Co_3_O_4_/3C-SiC photoanode was prepared following the procedure illustrated in Figure S1. Our group has previously demonstrated the successful growth of high-quality n-type 3C-SiC films using the sublimation technique.^15−17^ The X-ray diffraction (XRD) pattern of the synthesized 3C-SiC shows two distinct diffraction peaks (Figure S2), corresponding to the (111) and (222) reflections of 3C-SiC. The high-resolution XRD (HRXRD) ω-scan rocking curve of the (111) Bragg reflections reveal a symmetric rocking curve with a full width at half-maximum (fwhm) value of 34 arcseconds, indicating the high crystalline quality of the 3C-SiC samples.^18^Figure 1a presents scanning electron microscopy (SEM) image of pristine 3C-SiC, showing a smooth surface morphology. The ultrasmall colloidal Co_3_O_4_ nanoparticles were synthesized using the hydrothermal method.^19^ Dynamic light scattering (DLS) measurements were conducted to assess the size distribution of the Co_3_O_4_ nanoparticles in the colloidal solution, revealing a uniform size of approximately 5 nm (Figure S3). Transmission electron microscopy (TEM) analysis showed a particle size of 4.74 ± 1.20 nm (Figure 1b and Figure S4), while high-resolution TEM (HRTEM) revealed lattice fringes of 0.241 nm, corresponding to the (311) plane of Co_3_O_4_ (Figure 1c).^20^

XRD analysis confirmed the crystal structure of the synthesized Co_3_O_4_ nanoparticles, which matched the data from JCPDS card No. 43–1003 (Figure S5).^21^ The Co_3_O_4_/3C-SiC p-n heterojunctions were fabricated by depositing the colloidal solution onto the 3C-SiC substrates, followed by thermal treatment (details in the Experimental Section). As shown in Figure 1d-e, the Co_3_O_4_ nanoparticles were successfully deposited on the 3C-SiC surface, forming a 600 nm-thick Co_3_O_4_ layer. After depositing the Ni(OH)_2_ layer as the OEC, the Ni(OH)_2_/Co_3_O_4_/3C-SiC photoanode exhibited a dense layer of nanoparticles (Figure 1f). Energy Dispersive Spectrometry (EDS) mapping of Co and Ni in the photoanode confirmed the successful deposition of Co_3_O_4_ and Ni(OH)_2_ onto 3C-SiC (Figure 1g-i).

**Figure 2:**
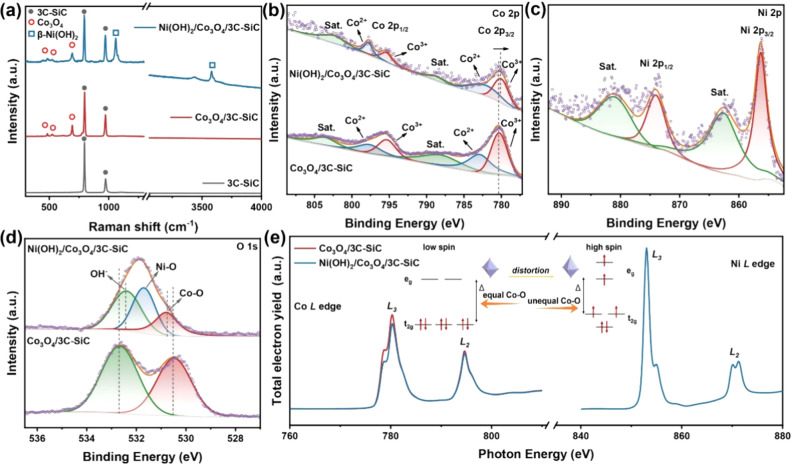
(a) Raman spectra of 3C-SiC, Co_3_O_4_/3C-SiC, and Ni(OH)_2_/Co_3_O_4_/3C-SiC. (b) XPS Co 2p spectra of Co_3_O_4_/3C-SiC and Ni(OH)_2_/Co_3_O_4_/3C-SiC. (c) XPS Ni 2p spectra of Ni(OH)_2_/Co_3_O_4_/3C-SiC. (d) XPS O 1s spectra of Co_3_O_4_/3C-SiC and Ni(OH)_2_/Co_3_O_4_/3C-SiC. (e) sXAS spectra of Co *L* edge of Co_3_O_4_/3C-SiC and Ni(OH)_2_/Co_3_O_4_/3C-SiC, and Ni *L* edge of Ni(OH)_2_/Co_3_O_4_/3C-SiC.

To further investigate the structural properties of the synthesized materials, we performed Raman spectroscopy on 3C-SiC, Co_3_O_4_/3C-SiC, and Ni(OH)_2_/Co_3_O_4_/3C-SiC, as shown in Figure 2a. The Raman spectrum of 3C-SiC displayed its characteristic transverse and longitudinal optical phonon modes, which were observed in all samples. For Co_3_O_4_/3C-SiC, prominent peaks at 482 cm^–1^, 520 cm^–1^, and 690 cm^–1^, corresponded to the E_g_ mode, F_2g_ mode, and A_1g_ mode, confirming the spinel structure of Co_3_O_4_.^22^ The Ni(OH)_2_/Co_3_O_4_/3C-SiC sample showed distinct peaks at 1060 cm^–1^ and 3578 cm^–1^, corresponding to vibrational modes associated with metal–oxygen bonding and hydroxyl groups, respectively.^23,24^ These results confirm the successful formation of β-Ni(OH)_2_ on the Co_3_O_4_/3C-SiC surface.

We further explored the interfacial interactions in the β-Ni(OH)_2_/Co_3_O_4_/3C-SiC system using XPS, soft X-ray absorption spectroscopy (sXAS), X-ray absorption near-edge structure spectroscopy (XANES), and extended X-ray absorption fine structure spectroscopy (EXAFS). Figure 2b shows the Co 2p spectra for Co_3_O_4_/3C-SiC and Ni(OH)_2_/Co_3_O_4_/3C-SiC. Two primary doublets corresponding to the Co^2+^ and Co^3+^ states are observed. Notably, the Co 2p_3/2_ peak for Ni(OH)_2_/Co_3_O_4_/3C-SiC is slightly shifted to a lower binding energy compared to Co_3_O_4_/3C-SiC, indicating increased electronic delocalization of Co ions. This shift further confirms the existence of electron transfer from Ni(OH)_2_ to Co_3_O_4_ due to the strong electronic coupling at the heterointerface.^25^ The Ni 2p spectrum of Ni(OH)_2_/Co_3_O_4_/3C-SiC showed two main peaks at 874.0 and 856.3 eV, corresponding to Ni 2p_3/2_ and Ni 2p_1/2_, along with their satellite peaks (Figure 2c).^26^Figure 2d presents the O 1s XPS spectra of Co_3_O_4_/3C-SiC and Ni(OH)_2_/Co_3_O_4_/3C-SiC. For Co_3_O_4_/3C-SiC, the O 1s peaks were attributed to Co–O bonds (530.5 eV) and adsorbed oxygen species (532.6 eV). In the Ni(OH)_2_/Co_3_O_4_/3C-SiC sample, the broad band is deconvoluted into three components: Ni–O (531.7 eV), Co–O (530.7 eV), and adsorbed oxygen (532.4 eV). Compared to Co_3_O_4_/3C-SiC, the slight shift of the Co–O peak toward higher binding energies in Ni(OH)_2_/Co_3_O_4_/3C-SiC is attributed to the formation of Ni–O bonds and Ni–O–Co bonds at the Ni(OH)_2_/Co_3_O_4_ interface, indicating strong electronic interactions between the layers.

Moreover, sXAS was used to investigate the local electronic environment of structure, including the distinction of orbital occupation and spin state differences among samples. The Co *L*-edge and Ni *L*-edge spectra are presented in Figure 2e. The Ni *L*-edge spectrum, resulting from the dipole-allowed 2p→3d transitions, showed two distinct features separated by ∼17 eV, corresponding to *L*_3_ (2p_3/2_→3d transitions) and *L*_2_ (2p_1/2_→3d transitions).^27,28^ These results confirm the successful formation of Ni(OH)_2_, as supported by Raman and XPS data.

The Co *L*-edge spectrum, arising from Co 2p→3d transition, similarly displayed *L*_3_ and *L*_2_ edges due to spin–orbital coupling, with the *L*_3_ edge influenced by the interaction between Co 3d orbitals and O 2p orbitals.^29,30^ The spectral shape, particularly for the *L*_3_ edge at lower photon energies, is significantly influenced by the complex interactions among Co 3d orbitals and their hybridization with ligand O 2p orbitals. Therefore, the shape and normalized intensity of the *L*_3_ edge peak provide insights into the orbital occupancy and spin states of Co atoms.^31^

In the Ni(OH)_2_/Co_3_O_4_/3C-SiC sample, the Co *L*_3_ edge peaks showed reduced intensity compared to those in Co_3_O_4_/3C-SiC, indicating increased electron filling in the Co e_g_ orbitals. The deposition of Ni(OH)_2_ on Co_3_O_4_ forms a heterostructure interface with Ni–O–Co bonds, which distorts the octahedral structure of Co_3_O_4_. This distortion induces a spin-state transition in cobalt, shifting from low-spin states, where electrons primarily occupy the t_2g_ orbitals, to high-spin states with partial filling in the e_g_ orbitals, as illustrated in Figure 2e.^32^ These high-spin states are known to improve the OER activity in Co_3_O_4_.^33−36^

**Figure 3:**
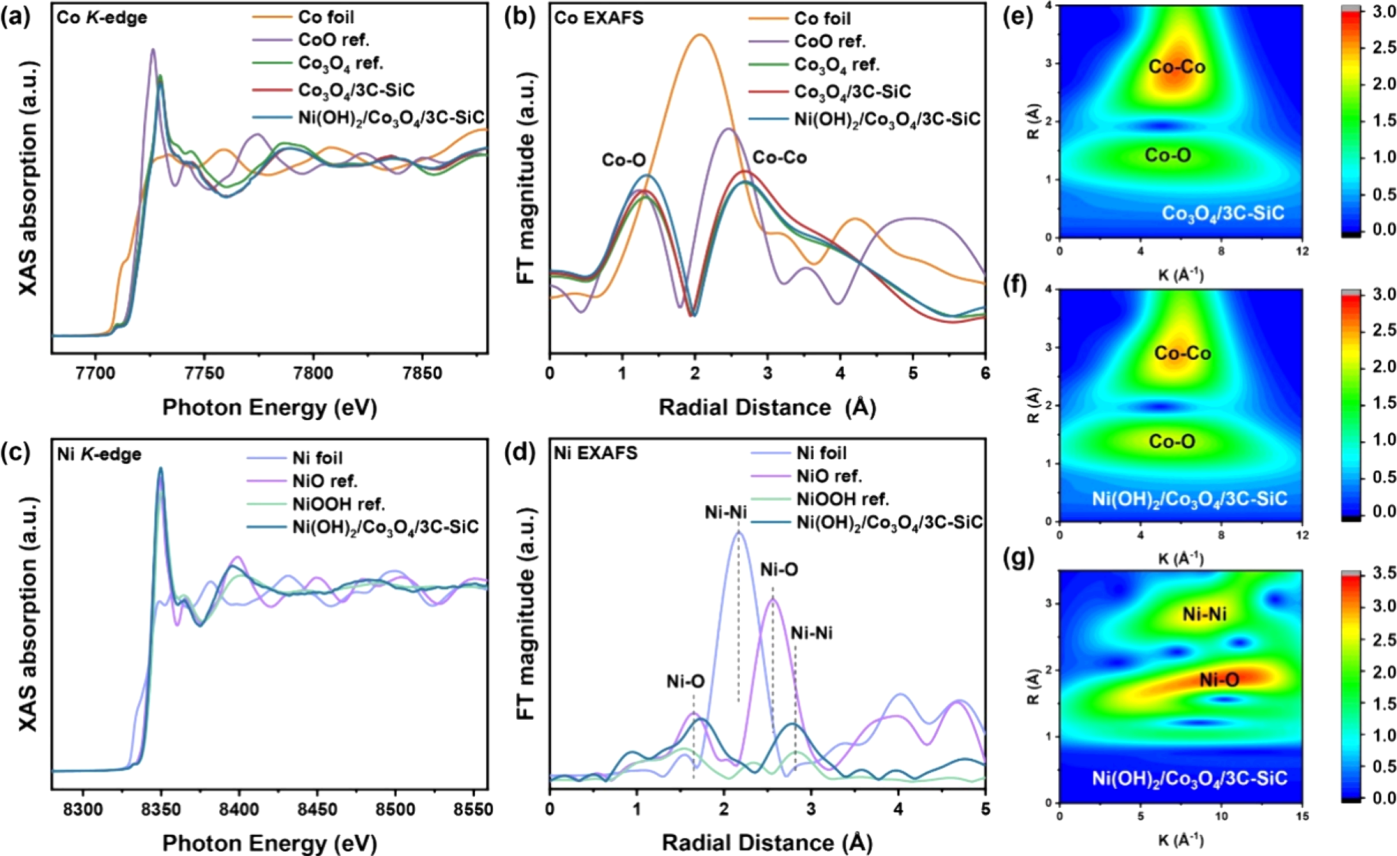
(a) Co *K* edge XANES; (b) Co *K* edge EXAFS; (c) Ni *K* edge XANES; (d) Ni *K* edge EXAFS; The corresponding WT for the k^3^-weighted Co *K* edge EXAFS signals of (e) Co_3_O_4_/3C-SiC, and (f) Ni(OH)_2_/Co_3_O_4_/3C-SiC. (g) The corresponding WT for the k^3^-weighted Ni *K* edge EXAFS signals of Ni(OH)_2_/Co_3_O_4_/3C-SiC.

To gain further insights into the electronic structure and coordination environment of the samples, XANES and EXAFS spectroscopy were performed. Figure 3a shows the Co *K*-edge-normalized XANES spectra of Co foil, Co_3_O_4_ reference, Co_3_O_4_/3C-SiC, and Ni(OH)_2_/Co_3_O_4_/3C-SiC. The absorption edge of Ni(OH)_2_/Co_3_O_4_/3C-SiC shifts to a lower energy compared to Co_3_O_4_/3C-SiC, indicating an increase in electron delocalization with the deposition of Ni(OH)_2_ (Figure S6a).^37^ This is further supported by the lower white line peak energy in the Co *K*-edge XANES for Ni(OH)_2_/Co_3_O_4_/3C-SiC (Figure S6b), suggesting a reduced Co valence state due to dual-interface engineering, consistent with XPS results.^38^ Typically, the intensity of the white line is proportional to the density of d-orbital states, which is influenced by oxidation states and electron configurations.^39,40^ The lower white line intensity suggests a CoO_6_ octahedron with greater distortion and more occupied d-orbital states,^41^ which align with the Co *L*-edge results.

The Fourier transform of Co *K*-edge EXAFS spectra (FT-EXAFS) in R-space shows two prominent signals, corresponding to Co–O and Co–Co scattering, indicating that the bulk coordination structure of Co_3_O_4_ remains unchanged after the deposition of Ni(OH)_2_ (Figure 3b). However, the average Co–O band distance increases in Ni(OH)_2_/Co_3_O_4_/3C-SiC, indicating a lower oxidation state (Figure S7a). The bond elongation enhances electron interaction between Co and O, lowering the Co oxidation state.^42,43^ This elongated Co–O bond further supports the high-spin state Co in Ni(OH)_2_/Co_3_O_4_/3C-SiC.^33^ In addition, the intensity of the Co–Co key position in Ni(OH)_2_/Co_3_O_4_/3C-SiC decreases after the deposition of Ni(OH)_2_, indicating a loss of long-range order degree of the octahedral structure, likely due to the distortion in the CoO_5_ tetragonal cone and CoO_6_ octahedron at the interface (Figure S7b).^44^ Ni *K*-edge XANES analysis shows that the absorption edge of Ni(OH)_2_/Co_3_O_4_/3C-SiC lies between the edges for NiO and NiOOH references (Figure 3c and Figure S8), with the edge position closely matching that of Ni(OH)_2_. The FT-EXAFS spectrum of Ni(OH)_2_/Co_3_O_4_/3C-SiC shows two peaks at 1.75 and 2.56 Å (Figure 3d), corresponding to Ni–O and Ni–Ni correlations.^45^ These results further confirm the presence of Ni(OH)_2_ in the synthesized sample, consistent with Raman and XPS results.

Wavelet transform (WT)-EXAFS results reveal a significant positive shift in the main Co–O intensity for Ni(OH)_2_/Co_3_O_4_/3C-SiC compared to Co_3_O_4_/3C-SiC (Figure 3e, 3f, and Figure S9). This shift in the k value indicates electron delocalization through the Ni–O–Co chemical bonds, consistent with the results above. Furthermore, the WT-EXAFS of Ni(OH)_2_/Co_3_O_4_/3C-SiC displays two peaks at 1.76 and 2.57 Å on the R-axis, corresponding to Ni–O paths and the first shell of the Ni–Ni path, respectively (Figure 3g and Figure S10).^46,47^

**Figure 4:**
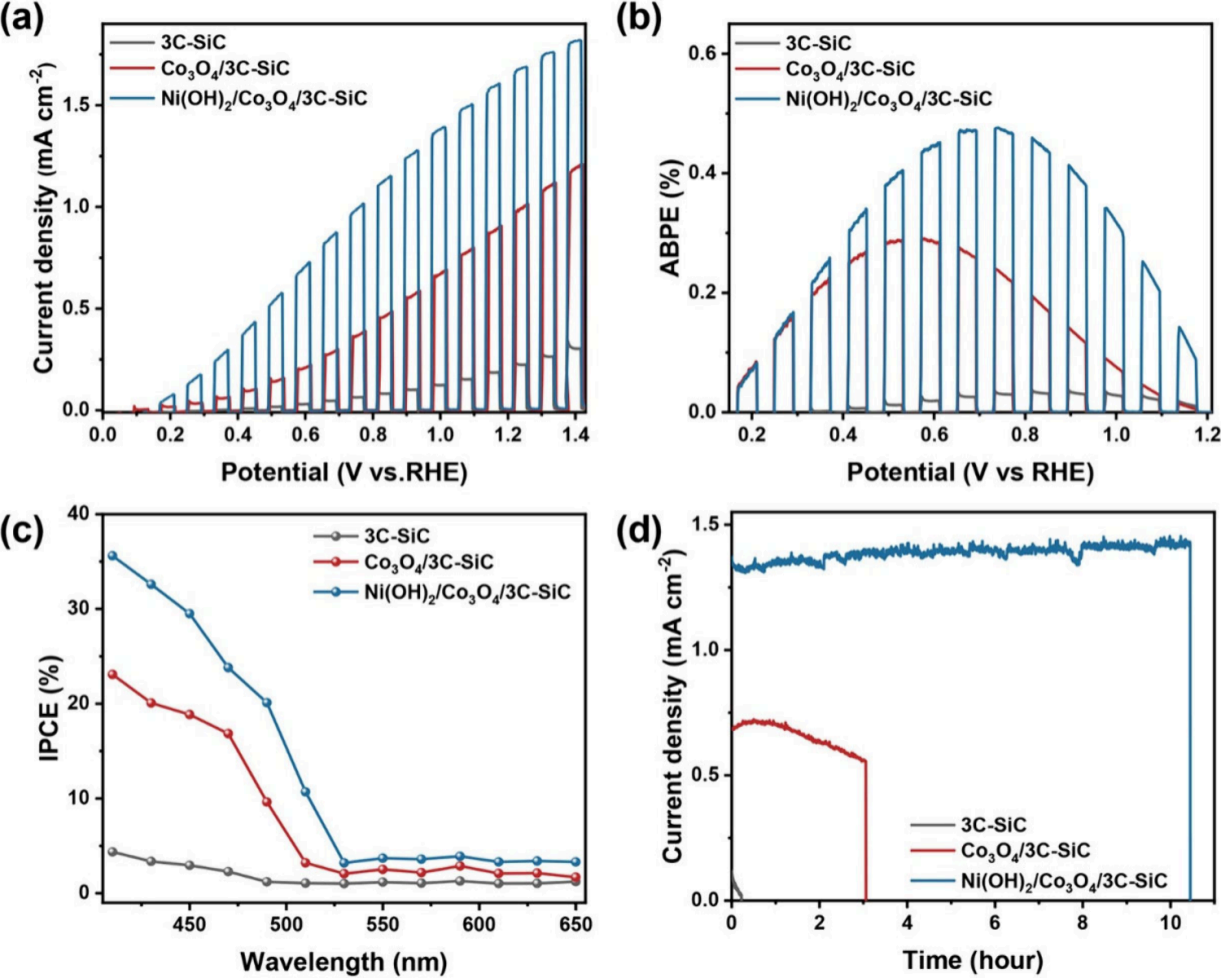
(a) Photocurrent density-potential curves, and (b) ABPE curves of 3C-SiC, Co_3_O_4_/3C-SiC, and Ni(OH)_2_/Co_3_O_4_/3C-SiC photoanodes measured in 1 M NaOH under chopped AM 1.5G, 100 mW cm^–2^ illumination, (c) IPCE of the photoanodes measured in 1.0 M NaOH at 1.0 V_RHE_. (d) Measured chronoamperometry as stability tests of the 3C-SiC, Co_3_O_4_/3C-SiC, and Ni(OH)_2_/Co_3_O_4_/3C-SiC photoanodes in 1.0 M NaOH at 1.0 V_RHE_ under steady-state AM1.5G 100 mW cm^–2^ illumination.

To explore the impact of dual interfacial engineering on the PEC water oxidation performance, we conducted PEC measurements using the synthesized samples as photoanodes. As illustrated in Figure 4a, the Ni(OH)_2_/Co_3_O_4_/3C-SiC photoanode achieved a high photocurrent density of 1.68 mA cm-^2^ at 1.23 V vs RHE, which is nearly 8-fold times higher than that of pristine 3C-SiC (0.22 mA cm^–2^) and 1.7 times greater than that of Co_3_O_4_/3C-SiC (0.98 mA cm^–2^), demonstrating the substantial enhancement in PEC activity achieved through dual interfacial engineering.

Notably, the optimized Ni(OH)_2_/Co_3_O_4_/3C-SiC photoanode outperformed previously reported systems, including the NiO/3C-SiC p–n junction photoanode and other cocatalyst/3C-SiC configurations such as NiFeOOH/3C-SiC and FeOOH/Graphene/3C-SiC (Table S1). These results emphasize the effectiveness of this dual-interface design. As revealed by the advanced spectroscopic analyses (XPS, sXAS, XANES, and EXAFS) above, the dual-interface engineering not only harnesses the synergistic role of Co_3_O_4_ and Ni(OH)_2_ layers but also induces fundamental modifications to the interfacial electronic structure. These modifications significantly enhance charge transport and catalytic activity. Specifically, the Co_3_O_4_ layer facilitates efficient extraction of photogenerated holes from 3C-SiC, transferring them to the Ni(OH)_2_ cocatalyst layer through interfacial Ni–O–Co bonds. This interfacial architecture accelerates oxygen evolution reaction (OER) kinetics, as demonstrated by the observed performance improvements.

Crucially, this dual-interface engineering transcends traditional structural optimization by introducing tailored modifications to the interfacial electronic structure, as illustrated in Figures 2 and 3. These electronic structure modifications promote efficient charge transfer and enhance catalytic activity. The synergistic combination of structural and electronic modification shows the great potential of dual interfacial engineering in advancing PEC water splitting technologies.

The applied bias photon-to-current efficiency (ABPE) of the Ni(OH)_2_/Co_3_O_4_/3C-SiC photoanode reached 0.47% at 0.65 V vs RHE, which is 15.6 and 1.8 times higher than that of the 3C-SiC and Co_3_O_4_/3C-SiC photoanodes, respectively (Figure 4b). To further examine the effect of monochromatic light on photocurrent density, we performed incident photon-to-current conversion efficiency (IPCE) measurements. The IPCE values for the Ni(OH)_2_/Co_3_O_4_/3C-SiC exceeded over 30% in the 350–450 nm range, significantly surpassing the values for 3C-SiC and Co_3_O_4_/3C-SiC (Figure 4c). These results are consistent with the enhanced photocurrent.

In addition to its high PEC activity, the Ni(OH)_2_/Co_3_O_4_/3C-SiC photoanode demonstrates significantly improved stability during PEC water oxidation. As shown in Figure 4d, the pristine 3C-SiC photoanode exhibited a rapid decline in photocurrent within 10 min, indicating poor stability due to surface oxidation during PEC reaction. The Co_3_O_4_/3C-SiC photoanode initially showed an increase in photocurrent, attributed to the photothermal effect of the Co_3_O_4_ layer. This was followed by a decrease in photocurrent, most probably caused by the oxidation of the exposed 3C-SiC surface due to incomplete or nonuniform coverage of Co_3_O_4_. Notably, the Ni(OH)_2_/Co_3_O_4_/3C-SiC photoanode maintained stable PEC performance for over 10 h of continuous operation, highlighting its exceptional durability. This finding emphasizes the critical role of interfacial engineering in boosting both PEC efficiency and long-term stability. Furthermore, the SEM and XPS analyses of the Co_3_O_4_/3C-SiC and Ni(OH)_2_/Co_3_O_4_/3C-SiC photoanodes after the PEC measurements revealed no significant changes in structure or composition, as shown in Figures S13–15. This confirms the robust long-term stability and durability of the Ni(OH)_2_/Co_3_O_4_/3C-SiC photoanode during PEC operation.

The electrochemical impedance spectroscopy (EIS) was performed to investigate the charge transfer behavior of the photoanodes (Figure S16). The EIS results show that the Co_3_O_4_/3C-SiC photoanode exhibits a significantly lower charge transfer resistance compared to pristine 3C-SiC.^48^ Among all photoanodes, the Ni(OH)_2_/Co_3_O_4_/3C-SiC photoanode displayed the smallest charge transfer resistance (Table S2). These findings evidence that the Co_3_O_4_ layer greatly improves the transfer of photogenerated holes, while the Ni(OH)_2_ cocatalyst further facilitates efficient charge transfer to the electrolyte for boosting the oxygen evolution reaction.

**Figure 5:**
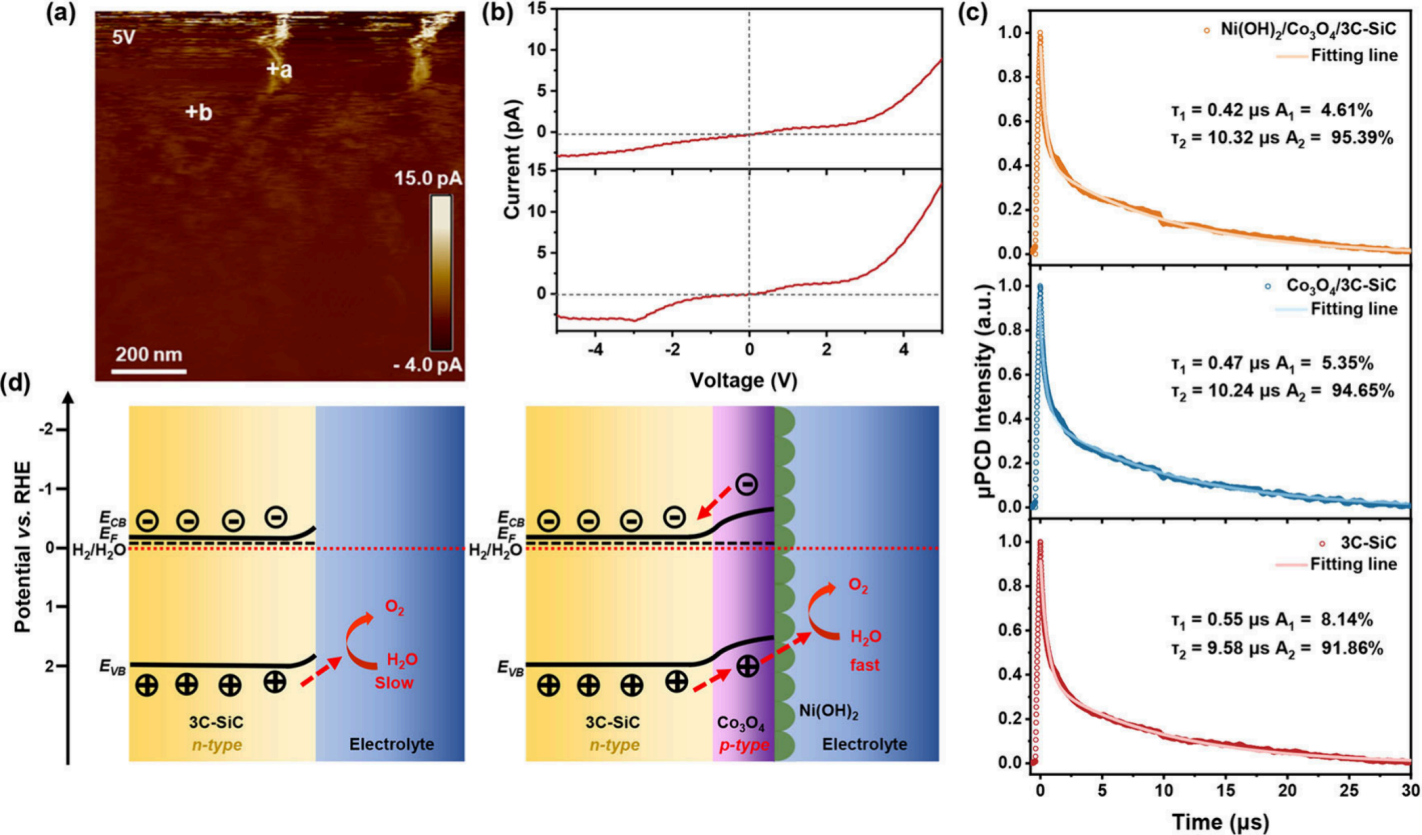
(a) C-AFM current map of the Co_3_O_4_/3C-SiC measured at a bias of 5 V. (b) The current–voltage (*I*–*V*) curves were measured at points a and b, as indicated in Figure 5a. (c) The microwave photoconductivity decay (μ-PCD) results of 3C-SiC, Co_3_O_4_/3C-SiC, and Ni(OH)_2_/Co_3_O_4_/3C-SiC. Schematic diagrams of the PEC mechanisms (d) 3C-SiC photoanodes and Ni(OH)_2_/Co_3_O_4_/3C-SiC photoanodes. E_F_: Fermi energy level; E_CB_: conduction band edge; E_VB_: valence band edge.

To confirm the formation of Co_3_O_4_/3C-SiC p–n junction, the conductive Atomic Force Microscopy (c-AFM) measurements were carried out. Figure S17 represents a typical AFM image of Co_3_O_4_/3C-SiC p–n junction. As shown in Figure 5a, the c-AFM current map at a bias of 5 V indicates uniform conductivity across the sample, which is essential for efficient charge transport. The current–voltage (*I*–*V*) characteristics depicted in Figure 5b exhibit typical diode-like rectifying behavior, providing strong evidence for the successful formation of the Co_3_O_4_/3C-SiC p-n heterojunction.

To investigate charge carrier dynamics and their impact on PEC water-splitting performance, we assessed the minority carrier lifetime of the 3C-SiC, Co_3_O_4_/3C-SiC, and Ni(OH)_2_/Co_3_O_4_/3C-SiC photoanodes using μ-PCD measurements. Figure 5c shows that the decay curves for all samples fit well with a biexponential model, consisting of a fast-decay component (τ_1_) and a slow-decay component (τ_2_). Previous works indicate that τ_1_ represents recombination at the surface or interface while τ_2_ reflects the carrier lifetime within the bulk of 3C-SiC.^15^

Notably, the bulk carrier lifetime τ_2_ predominantly dominates the decay curve for all three samples. The pristine 3C-SiC exhibited a bulk carrier lifetime τ_2_ of 9.58 μs (contributing 91.86% to the decay curve). The Co_3_O_4_/3C-SiC increased τ_2_ to 10.24 μs (94.65%). The Ni(OH)_2_/Co_3_O_4_/3C-SiC further enhanced τ_2_ to 10.32 μs (95.39%). The improvement in Co_3_O_4_/3C-SiC is attributed to the built-in electric field of p–n junction, which enhances spatial charge separation and thus reduces charge recombination.^49^ The additional increase in τ_2_ for Ni(OH)_2_/Co_3_O_4_/3C-SiC results from dual-interface engineering, where Ni–O–Co bond formation modulates the electronic structure, enhances charge transfer, and suppresses recombination losses, leading to enhanced PEC performance.^50^ Furthermore, the orbital hybridization and spin-state tuning induced by these bonds further reduce the likelihood of fast recombination and optimize charge transport.^51,52^

The formation of Co_3_O_4_/3C-SiC p–n junction is further confirmed by the Mott–Schottky plots measurements. As shown in Figure S18, the positive slope of the Mott–Schottky plot for 3C-SiC confirms its n-type nature, while the negative slope for Co_3_O_4_ indicates its p-type semiconductor characteristics. The flat band potentials of 3C-SiC and Co_3_O_4_ were determined to be −0.22 and 1.51 V versus RHE, respectively. It is important to note that the flat band potential represents the Fermi level of the semiconductor relative to the potential of the reference electrode when band bending is zero. When the n-type 3C-SiC contacts the p-type Co_3_O_4_, electrons diffuse from 3C-SiC into Co_3_O_4_, while holes move in the opposite direction until the Fermi levels of both semiconductors align. This process results in the formation of a p–n junction with a built-in potential at the Co_3_O_4_/3C-SiC interface.^53^ From the difference in Fermi levels between 3C-SiC and Co_3_O_4_ prior to contact, the built-in potential at the interface of the Co_3_O_4_/3C-SiC p–n junction is estimated to be 1.73 V. Figure 5d illustrate the energy band diagrams for 3C-SiC and Ni(OH)_2_/Co_3_O_4_/3C-SiC photoanodes during photoelectrochemical water splitting. The Co_3_O_4_/3C-SiC p–n junction generates a built-in electric field, creating a strong driving force that facilitates the efficient separation and transfer of charge carriers.

## Conclusions

In conclusion, this study demonstrates the pivotal role of dual-interface engineering and electronic structure regulation in enhancing the PEC performance of Ni(OH)_2_/Co_3_O_4_/3C-SiC photoanodes. By integrating Co_3_O_4_ as an efficient hole-extraction layer to form a p-n heterojunction with 3C-SiC, and employing Ni(OH)_2_ as a cocatalyst, we achieved substantial improvements in PEC water oxidation performance. Advanced spectroscopic analyses (XPS, sXAS, XANES, and EXAFS) and μ-PCD measurements revealed that dual-interface engineering not only enhances interfacial electronic coupling but also prolongs minority carrier lifetimes, demonstrating the critical connection between electronic structure modulation, charge separation efficiency, and enhanced PEC performance. These synergistic structural and electronic enhancements significantly improve charge transport and accelerate oxygen evolution reaction kinetics. These findings represent a significant advancement in the field of PEC water splitting, paving the way for next-generation semiconductor-based solar energy devices.

## Supporting Information Available

Experimental and computational details, characterizations, and additional experimental results. (PDF)
