# Supplementary material for: Manipulating Electron Structure through Dual-Interface Engineering of 3C-SiC Photoanode for Enhanced Solar Water Splitting
Source: J Am Chem Soc. 2025 Apr 17;147(17):14815–23. doi: 10.1021/jacs.5c04005 (PMC12046598; doi:10.1021/jacs.5c04005)
Supplement: Supplementary file 1 — ja5c04005_si_001.pdf [file ja5c04005_si_001.pdf]

Supporting Information

Manipulating Electron Structure through Dual-Interface Engineering of 3C-SiC Photoanode for Enhanced Solar Water Splitting

Hui Zeng,[1] Satoru Yoshioka,[2] Weimin Wang,[3] Zhongyuan Han,[4] Ivan G Ivanov,[1] Hongwei Liang,[4] Vanya Darakchieva,[1,5] and Jianwu Sun[1,*]

[1]Department of Physics, Chemistry and Biology (IFM), Linköping University, SE-58183, Linköping

[2]Department of Applied Quantum Physics and Nuclear Engineering, Kyushu University, Motooka 744, Nishi-ku, Fukuoka 819-0395, Japan

[3]MAX IV Laboratory, Fotongatan 2, SE-22484, Lund, Sweden

[4]School of Integrated Circuits, Dalian University of Technology, Dalian 116024, China

[5]NanoLund and Solid State Physics, Lund University, S-22100 Lund, Sweden

[*]Corresponding author. E-mail: jianwu.sun@liu.se

Experimental section

Preparation of the 3C-SiC Photoanode

All analytical grade chemicals are used as they were received without further purification. Through a sublimation process, 3C-SiC(111) with a thickness of around 1 mm was grown on a 4° off-axis n-type 4H-SiC.[1-4] To obtain single-crystal 3C-SiC with a thickness of around 300 μm, the 4H-SiC substrate and the interface layer between the 4H-SiC and 3C-SiC were carefully polished away. After that, the 3C-SiC(111) substrate was thoroughly cleaned with acetone, ethanol, $H_2O/NH_3/H_2O_2$ (5:1:1), $H_2O/HCl/H_2O_2$ (6:1:1), and hydrofluoric acid. Then, the backside of 3C-SiC was deposed in a 60 nm Ni/200 nm Au layer, followed by an annealing at 900 °C in N_2 for 10 minutes. After that, the 3C-SiC photoanode was sealed with epoxy resin, and only the 3C-SiC surface was exposed to the solution for light illumination.

Preparation of Co_3O_4 nanoparticle colloid solution

Co_3O_4 nanoparticles were synthesized by a hydrothermal method. 0.4 mL of ammonia solution was dissolved in 25 mL of ethanol solution containing 1 mM of cobalt acetate and stirred for 15 minutes. Then, the stirred solution was transferred to a 50 mL Teflon-lined stainless-steel autoclave and then kept in an oven at 120 ℃ for 1 h. After hydrothermal synthesis, the stainless-steel autoclave was cooled to room temperature in flowing water, and the obtained Co_3O_4 nanoparticle colloid solution was directly used in the following experiments without further modification.

Preparation of the Co_3O_4/3C-SiC Photoanode

The Co_3O_4 nanoparticle colloid solution (pH=10.5) was ultrasonicated for 30 min to maintain the uniform dispersion of the Co_3O_4 nanoparticles in the solution. A specific volume (50, 100, and 150 μL) of the uniform Co_3O_4 nanoparticle colloid solution was dripped on 3C-SiC films by a pipette, followed by completely drying at 80 ℃ for 30 min and then annealed at 450 ℃ in a Muffle furnace for 2 h. By changing the volume of the Co_3O_4 colloid solution, the loading amount can be controlled, optimizing the best PEC performance. The dipping volume of 100 μL gives the optimized PEC performance, as shown in Figure S11.

Preparation of the $Ni(OH)_2/Co_3O_4$/3C-SiC Photoanode

$Ni(OH)_2$ was photo-assisted electrodeposited from a 0.1 M $Ni(SO_4)_2 \cdot 6H_2O$ solution (pH=6.8-7.2, adjusting by 0.1 M KOH) at 0.11 V vs. Ag/AgCl for 20, 40, 60, and 80 s in a three-electrode cell. The best PEC performance can be optimized and obtained by changing the deposition time and controlling the $Ni(OH)_2$ loading amount. The deposition time of 40 seconds results in the optimized PEC performance, as shown in Figure S12.

Photoelectrochemical (PEC) measurements

PEC measurements were carried out on an electrochemical workstation (Princeton Applied Research, VersaSTAT3) at room temperature using a conventional three-electrode cell. The prepared photoanodes were employed as the work electrodes, while Pt and Ag/AgCl were used as the reference and cathode electrodes, respectively. The AAA solar simulator supplied the 100 mW cm^{-2} AM1.5G illumination light (LOT-Quantum Design GmbH).1 M NaOH solution (pH = 13.6) was used as the electrolyte.

All applied potentials versus Ag/AgCl electrode were converted to the potentials versus the reversible hydrogen electrode (RHE) using the Nernst equation below:

$$E_{RHE} = E_{Ag/AgCl} + 0.059\ pH + E^{\theta}_{Ag/AgCl}$$

where $E^{\theta}_{Ag/AgCl} = 0.1976V$ at 25 ℃.

Applied bias photon-to-current efficiency (ABPE) was calculated using the following equation:

$$ABPE = \frac{J\ (mA/cm^2) \times (1.23 - |V_{applied}|)\ (V)}{P\ (mW/cm^2)} \times 100\%,$$

where J is the photocurrent density at the applied bias $V_{applied}$, P is the incident illumination power density (100 mW cm^{-2}).

The incident-photon-to-current efficiency (IPCE) was obtained using the following equation:

$$IPCE\ (\%) = \frac{1240\ (V \times nm) \times J\ (mA/cm^2)}{P\ (mW/cm^2) \times \lambda\ (nm)} \times 100\%$$

where 1240 (V nm) represents a multiplication of h (Planck's constant) and c (the speed of light), J is the photocurrent density (mA/cm^2), λ is the incident light wavelength (nm), and P is the monochromatic illumination power intensity.

Electrochemical impedance spectroscopy (EIS) spectra were collected in a frequency range from 10 Hz to 100 kHz.

Characterization

X-ray diffraction (XRD) measurements were performed using a Philips MRD with Cu Kα radiation (λ = 1.54 Å). Scanning Electron Microscope (SEM) was carried out using Zeiss Sigma 300 and an energy-dispersive spectrometer (EDS). The transmission electron microscopy (TEM) high-resolution transmission electron microscopy

(HRTEM) was measured using the Tecnai G2 F20. The size distribution of nanoparticles was determined by Dynamic Light Scattering (DLS) (ZEN3690, Malvern Panalytical, UK). The surface morphology was characterized by an atomic force microscope (AFM) (Bruker Dimension XR, Bruker Corporation, USA) operating in tapping mode. The absorption spectra were measured with the UV-VIS spectrometer (Ocean Optics 2000+) and a stabilized tungsten-halogen light Source (Thorlabs, SLS201L).

The carrier lifetime was measured using the microwave photoconductivity decay (μ-PCD) system (MDPmap, Freiberg Instruments). A 355 nm laser with 10 ns pulse duration was used to excite samples, with a photon density of 1×10^{13} cm^{-2} on the sample surface. The photoconductivity decay was monitored by measuring microwave reflectivity, which decreases due to electron-hole pair recombination and thus indicates the carrier lifetime.

X-ray photoelectron spectroscopy (XPS) measurements were conducted by a PSP Vacuum Technology hemispherical electron-energy analyzer equipped with monochromated Al Kα radiation (hν = 1486.6 eV) at the beamline FinEstBeAMS in the MAX IV Laboratory synchrotron radiation laboratory, Lund, Sweden. XPS was measured on the as-prepared samples, i.e., with no Ar^+ etching. The soft X-ray absorption spectroscopy (sXAS) measurements were performed at the FinEstBeAMS beamline at the MAX IV synchrotron in Laboratory synchrotron radiation laboratory, Lund, Sweden. The X-ray photoelectron spectroscopy (XPS) and soft X-ray absorption spectroscopy (sXAS) measurements were performed at the Solid State End Station in

FinEstBeAMS beamline at the MAX IV synchrotron radiation laboratory, Lund, Sweden. XPS data were obtained by a SPECS Phoibos 150 R7 hemispherical electron-energy analyzer; the sXAS data were taken by drain current from sample as total electron yield.

The X-ray absorption spectroscopy (XAS) measurements were performed at Kyushu University beamline BL06 in Kyushu Synchrotron Light Research Center (SAGA-LS), Japan, using a Si(111) double-crystal monochromator. Co *K*-edge and Ni *K*-edge XAS spectra of the thin film samples were recorded in fluorescence yield mode using silicon drift detector. The reference specimens were diluted with high-purity hexagonal BN powder and measured in transmission geometry.

Supporting Figures and Tables

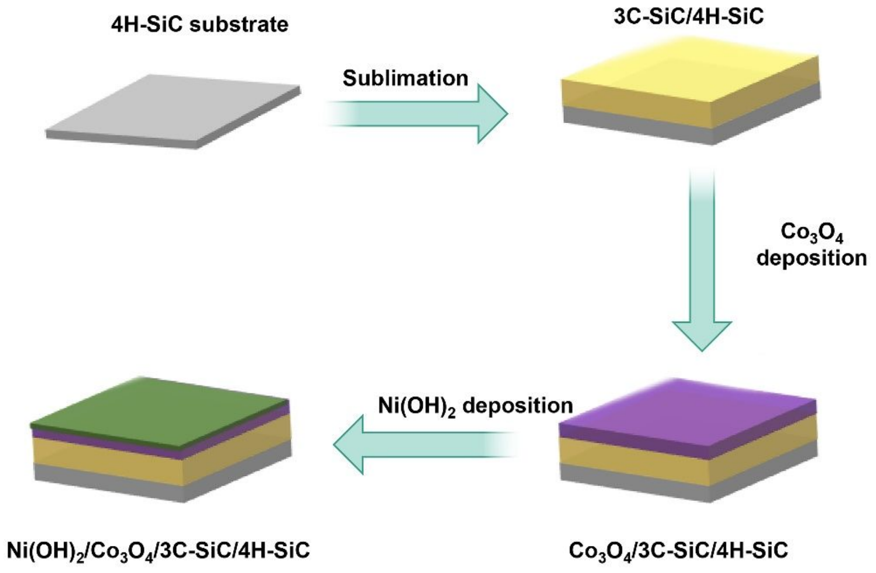

Supplementary Fig. 1. Schematic illustration of the synthesis procedure for the $Ni(OH)_2/Co_3O_4$/3C-SiC photoanode.

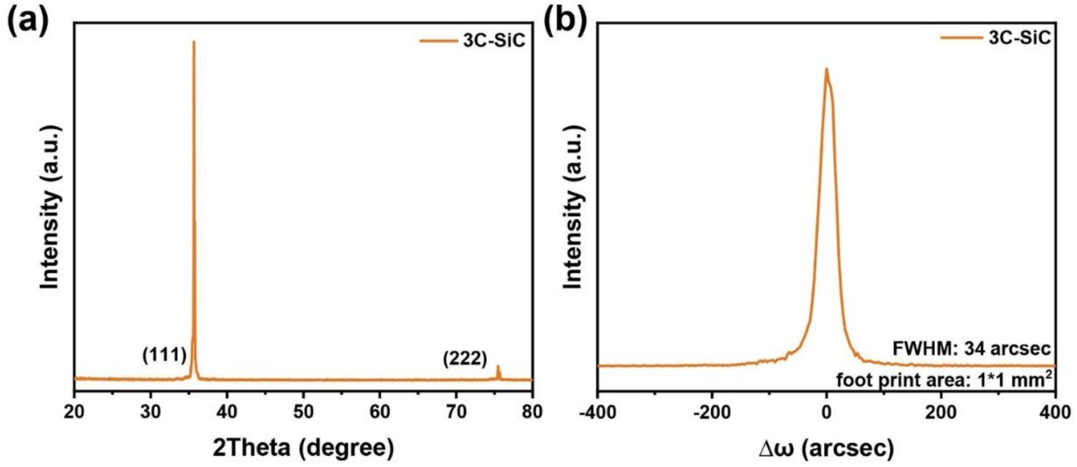

Supplementary Fig. 2. (a) XRD pattern of 3C-SiC films; (b) HRXRD ω-rocking curve of the 3C-SiC (111) reflection with a FWHM of 34 arcsec.

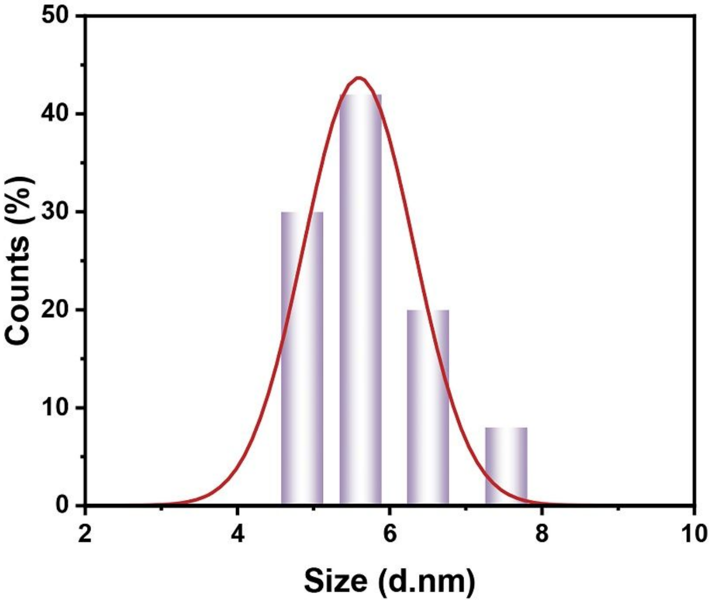

Supplementary Fig. 3. DLS of Co_3O_4 nanoparticles.

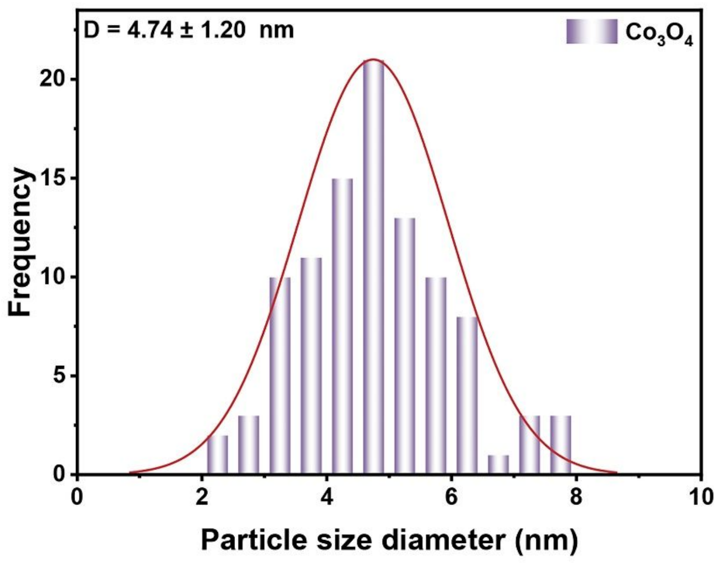

Supplementary Fig. 4. Particle size distribution analysis of Co_3O_4 nanoparticles from TEM measurements.

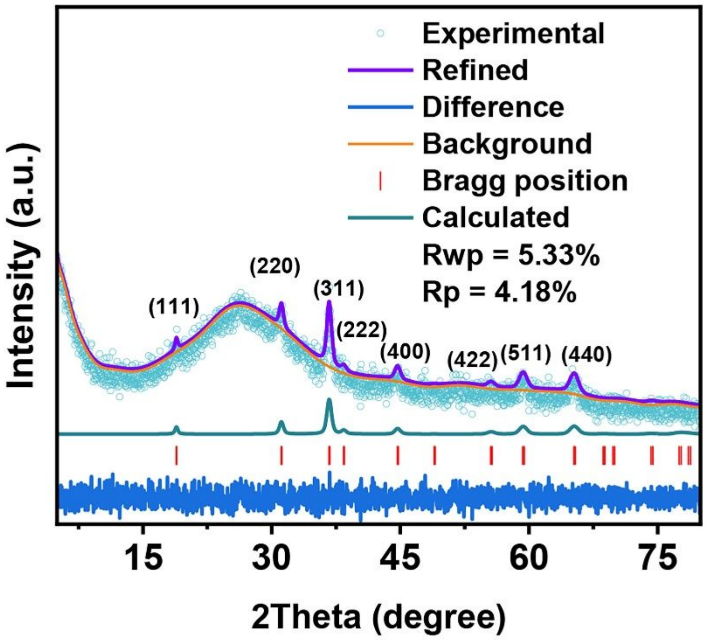

Supplementary Fig. 5. Rietveld refinement of XRD pattern of Co_3O_4 nanoparticles. The background is subtracted.

To collect enough Co_3O_4 samples for XRD characterization, 500 mL of Co_3O_4 colloid solution was prepared by repeating the hydrothermal reaction shown in the Experimental Section (Supporting Information) for 4 times (5 Teflon-lined stainless-steel autoclaves were used for one time), followed by evaporating the ethanol solvent in an oven at 80 ℃ overnight. XRD tested the obtained powder of Co_3O_4 nanoparticles.

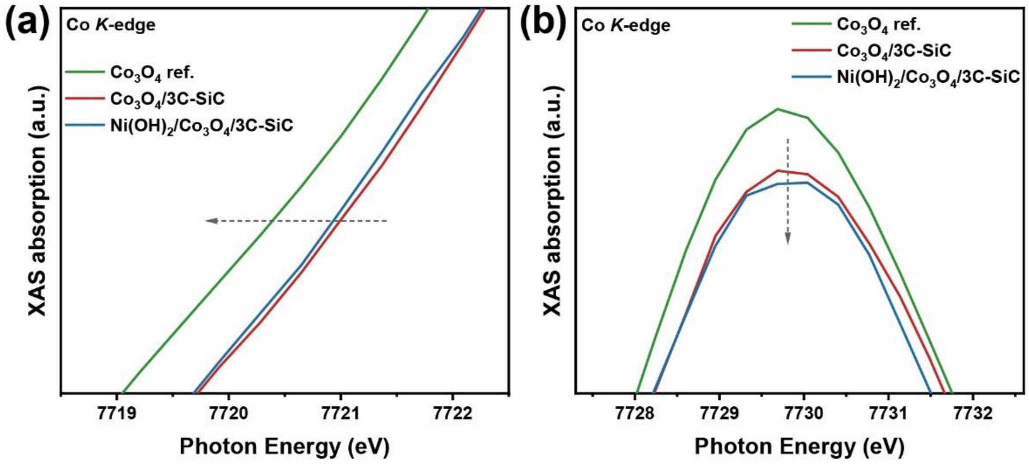

Supplementary Fig. 6. (a) Enlarged Co K edge XANES of the absorption edge; (b) Enlarged Co K edge XANES of the white line.

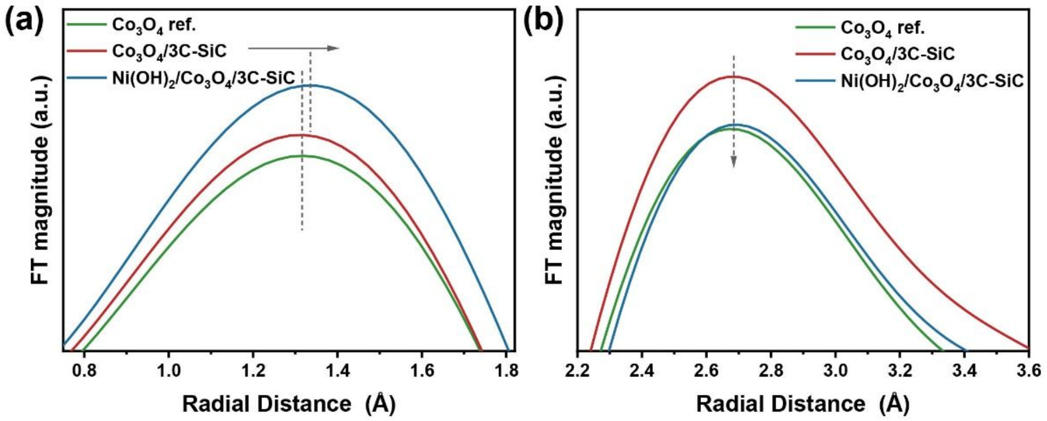

Supplementary Fig. 7. Enlarged Co K edge EXAFS.

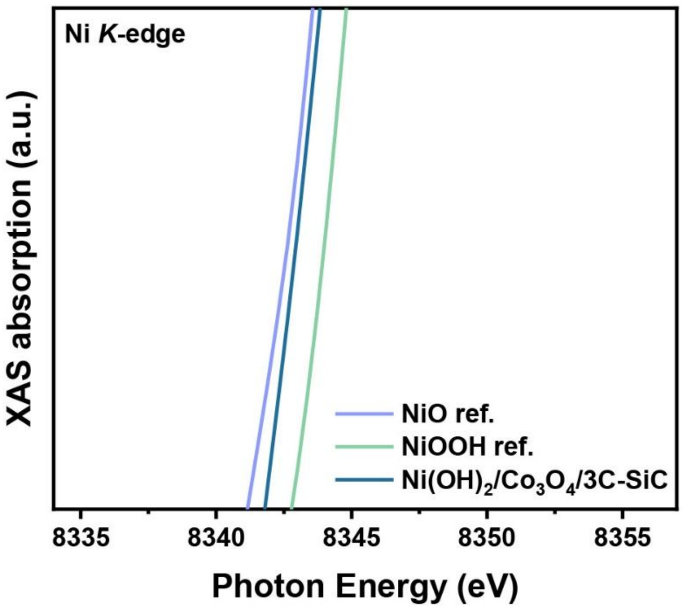

Supplementary Fig. 8. Enlarged Ni K edge XANES of the absorption edge.

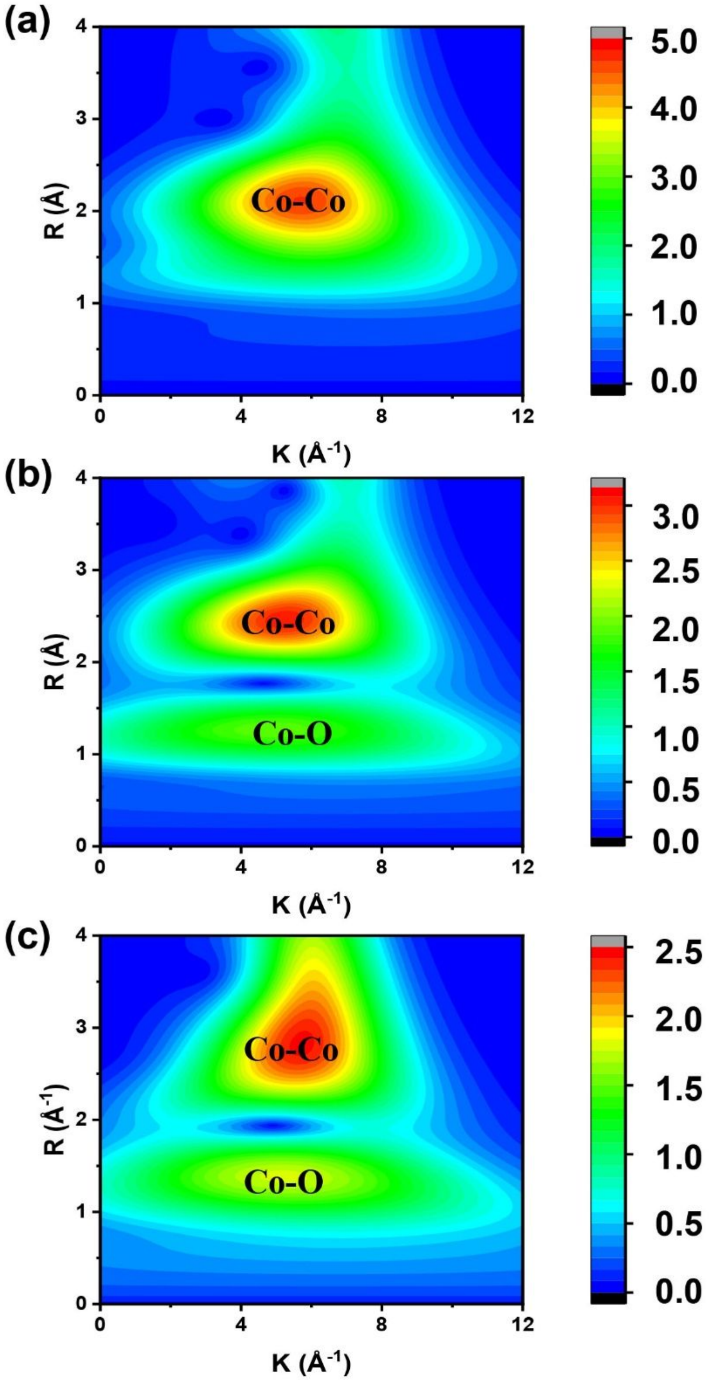

Supplementary Fig. 9. The corresponding WT for the k^3-weighted Co K edge EXAFS signals of Co foil, CoO reference, and Co_3O_4 reference.

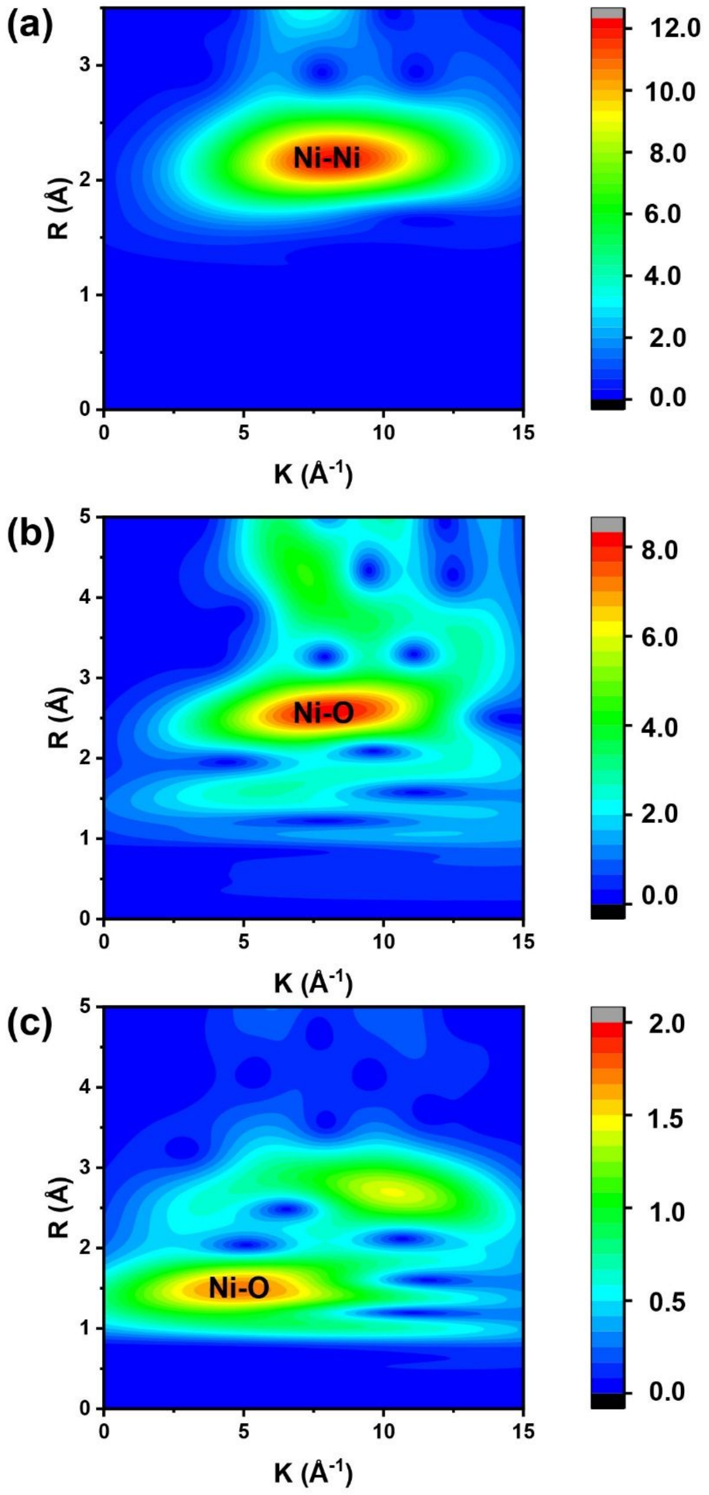

Supplementary Fig. 10. The corresponding WT for the k^3-weighted Ni K edge EXAFS signals of Ni foil, NiO reference, and NiOOH reference.

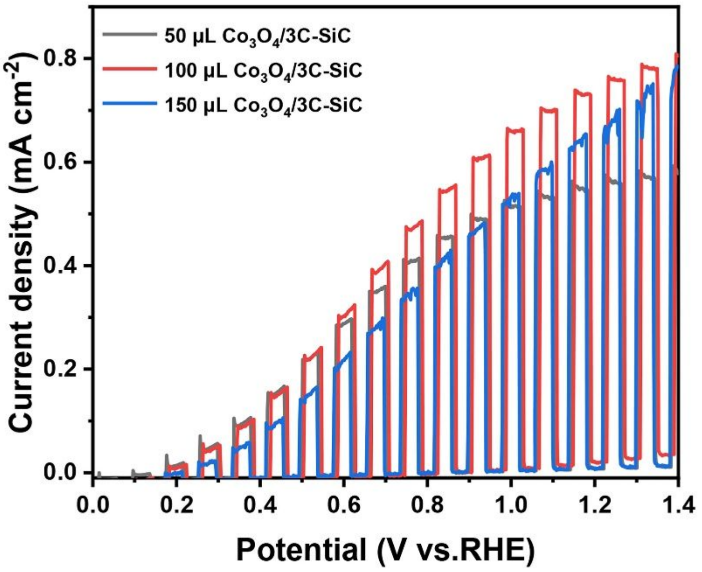

Supplementary Fig. 11. Current density-potential curves of the 3C-SiC and Co_3O_4/3C-SiC photoanodes with the different amounts of Co_3O_4, measured in 1.0 M NaOH under chopped AM1.5G 100 mW/cm^2 illumination.

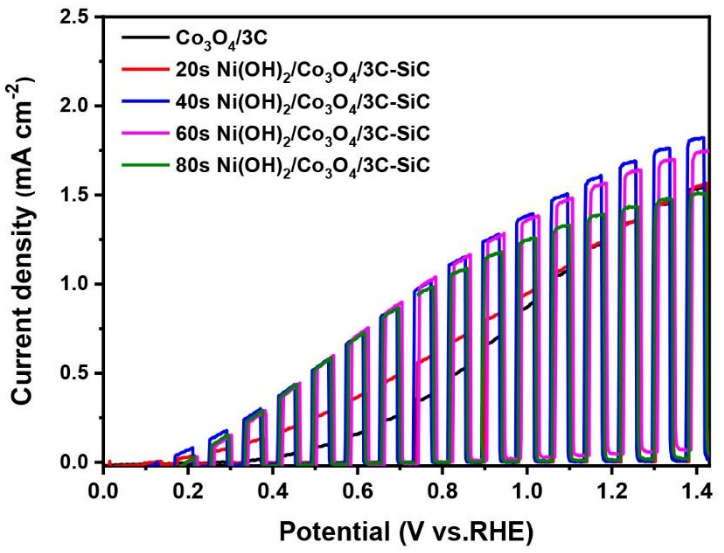

Supplementary Fig. 12. Current density-potential curves of the Co_3O_4/3C-SiC and $Ni(OH)_2$/Co_3O_4/3C-SiC photoanodes with photoelectrochemical deposition of $Ni(OH)_2$ for 20, 40, 60, and 80 seconds, measured in 1.0 M NaOH under chopped AM1.5G 100 mW/cm^2 illumination.

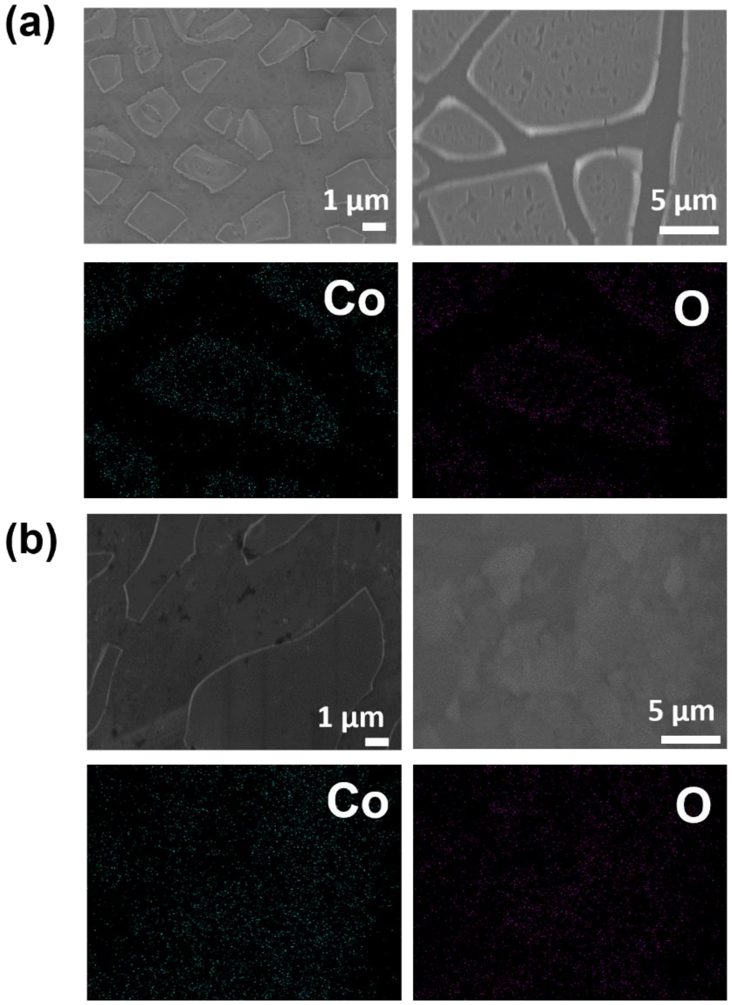

Supplementary Fig. 13. SEM image of Co_3O_4/3C-SiC photoanodes before (a) and after (b) reaction activity.

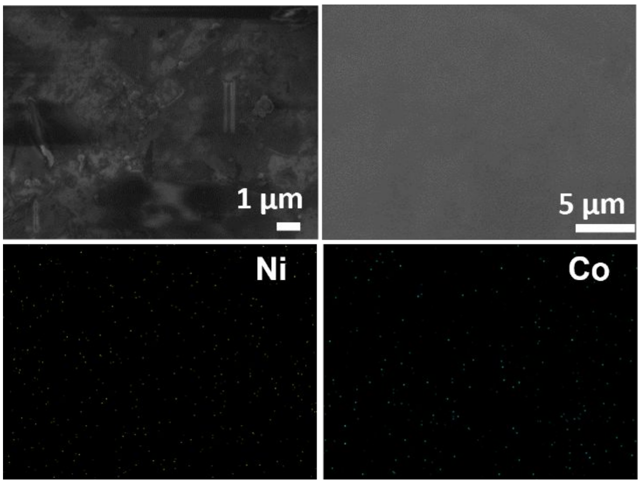

Supplementary Fig. 14. SEM image of $Ni(OH)_2/Co_3O_4$/3C-SiC photoanodes after reaction activity.

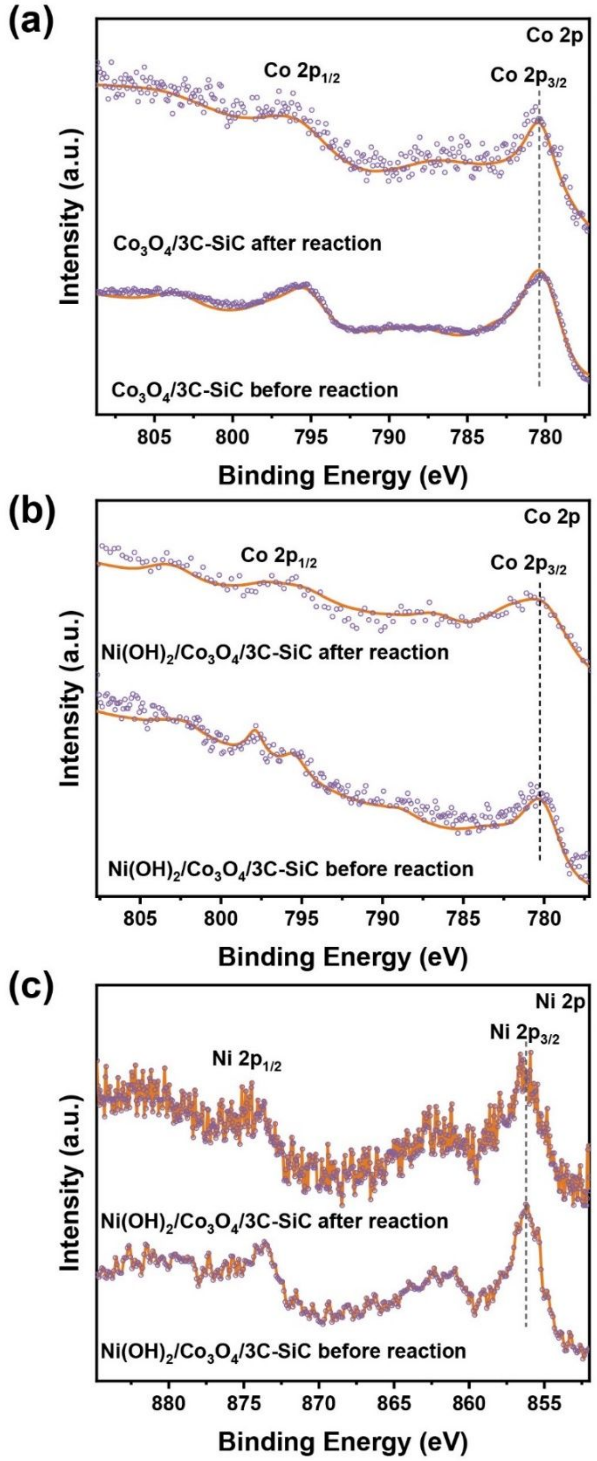

Supplementary Fig. 15. XPS Co 2p spectra of Co_3O_4/3C-SiC photoanodes before and after reaction activity (a); XPS Co 2p spectra of $Ni(OH)_2/Co_3O_4$/3C-SiC photoanodes before and after reaction activity (b); XPS Ni 2p spectra of $Ni(OH)_2/Co_3O_4$/3C-SiC photoanodes before and after reaction activity (c).

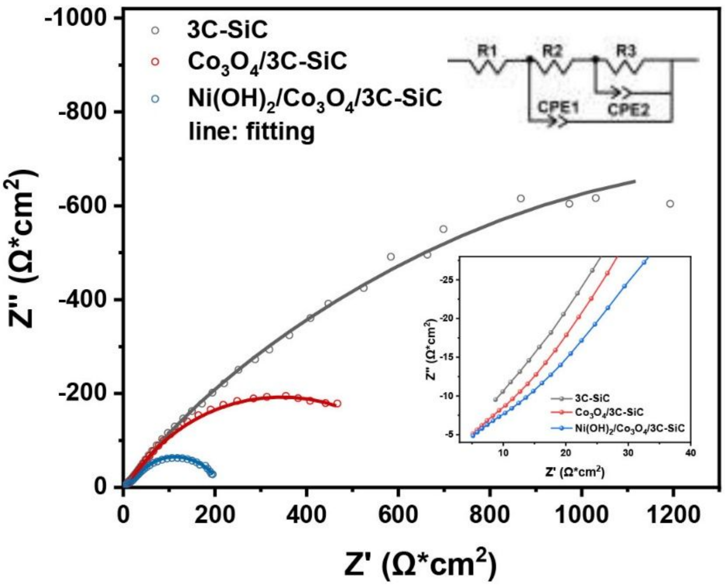

Supplementary Fig. 16. EIS spectra of 3C-SiC, Co_3O_4/3C-SiC, and $Ni(OH)_2$/Co_3O_4/3C-SiC photoanodes.

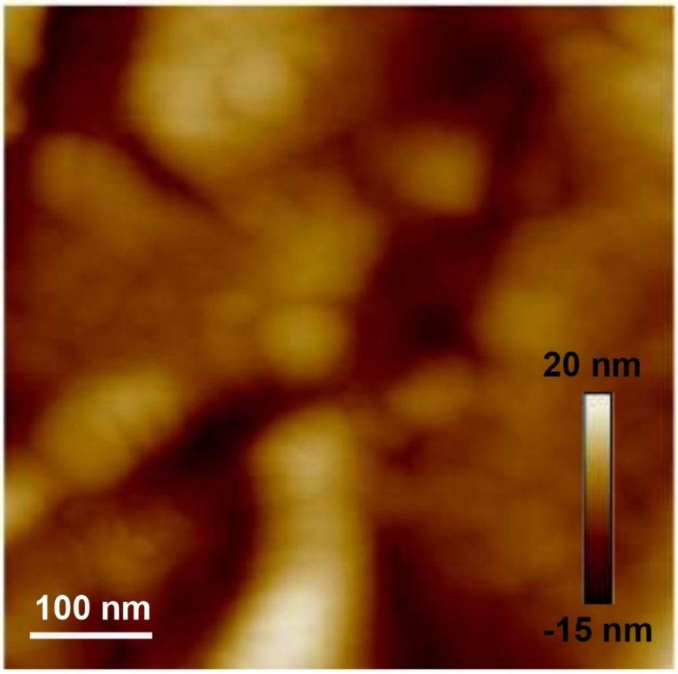

Supplementary Fig. 17. AFM image of Co_3O_4/3C-SiC p-n junction.

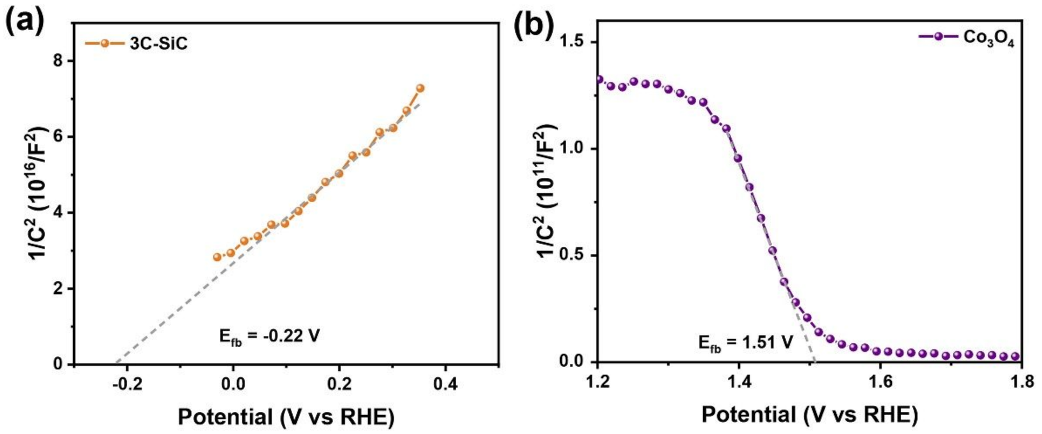

Supplementary Fig. 18. Mott-Schottky plots of (a) 3C-SiC and (b) Co_3O_4 in 1.0 M NaOH solution.

Supplementary Table 1. A recent comparison of the photocurrent densities of 3C-SiC photoanodes for PEC water splitting was reported.

Photoanodes	J vs. 1.23 V_{RHE}	PEC experimental conditions	Ref.
**3C-SiC/Co_3O_4**	**0.98 mA cm^{-2}**	**100 mW cm^{-2} AM1.5G; 1.0 M NaOH**	**This work**
**3C-SiC/Co_3O_4/$Ni(OH)_2$**	**1.68 mA cm^{-2}**	**100 mW cm^{-2} AM1.5G; 1.0 M NaOH**	**This work**
3C-SiC	~0.11 mA cm^{-2}	400 mW cm^{-2} Xe light; 0.1 M Na_2SO_4	5
3C-SiC	0.20 mA cm^{-2} at 1.0 V vs. Ag/AgCl	994 mW cm^{-2} Solar-light lamp; 0.01 M HCl	6
3C-SiC	0.13 mA cm^{-2} at 1.0 V vs. Ag/AgCl		
3C-SiC	0.70 mA cm^{-2} at 1.0 V vs. Ag/AgCl	100 mW cm^{-2} Xe lamp; 0.1 M $KHCO_3$	7
3C-SiC(111)	0.12 mA cm^{-2}	100 mW cm^{-2} AM1.5G; 1.0 M NaOH	8
3C-SiC/NiO	1.18 mA cm^{-2}	100 mW cm^{-2} AM1.5G; 1.0 M NaOH	4
3C-SiC(111)/FeOOH	0.73 mA cm^{-2}	100 mW cm^{-2} AM1.5G; 1.0 M NaOH	9
3C-SiC(111)/Ni:FeOOH	1.15 mA cm^{-2}	100 mW cm^{-2} AM1.5G; 1.0 M NaOH	
3C-SiC(111)/Monolayer graphene	0.67 mA cm^{-2}	100 mW cm^{-2} AM1.5G; 0.5 M $KHCO_3$	10
3C-SiC(111)/monolayer graphene/FeOOH	1.14 mA cm^{-2}	100 mW cm^{-2} AM1.5G; 0.5 M $KHCO_3$	
3C-SiC/NiFeOOH	0.69 mA cm^{-2}	100 mW cm^{-2} AM1.5G; 1.0 M NaOH	11

Supplementary Table 2. Fitting data of EIS measurements for 3C-SiC, Co_3O_4/3C-SiC, and $Ni(OH)_2/Co_3O_4$/3C-SiC under light of AM1.5G 100 mW/cm^2 illumination.

	**3C-SiC**	**Co_3O_4/3C-SiC**	**$Ni(OH)_2/Co_3O_4$/3C-SiC**
**R_s**	2.9	2.7	1.9
**R_{ct}**	40	26	11.3
**CPEsc-T**	5.9×10^{-7}	2.7×10^{-7}	3.1×10^{-7}
**CPEsc-P**	0.59	0.71	0.43
**$R_{ct,trap}$ (Ω cm^2)**	2003	622	198
**CPE_{trap}-T**	7.8×10^{-7}	5.9×10^{-7}	4.2×10^{-7}
**CPE_{trap}-P**	0.56	0.53	0.70

Supplementary Reference

(1) Sun, J. W.; Ivanov, I. G.; Liljedahl, R.; Yakimova, R.; Syväjärvi, M. Considerably long carrier lifetimes in high-quality 3C-SiC(111). *Appl. Phys. Lett.* **2012**, 100, 252101. DOI: 10.1063/1.4729583.

(2) Jokubavicius, V.; Yazdi, G. R.; Liljedahl, R.; Ivanov, I. G.; Yakimova, R.; Syväjärvi, M. Lateral Enlargement Growth Mechanism of 3C-SiC on Off-Oriented 4H-SiC Substrates. *Cryst. Growth Des.* **2014**, 14, 6514-6520. DOI: 10.1021/cg501424e.

(3) Jokubavicius, V.; Yazdi, G. R.; Liljedahl, R.; Ivanov, I. G.; Sun, J.; Liu, X.; Schuh, P.; Wilhelm, M.; Wellmann, P.; Yakimova, R.; et al. Single Domain 3C-SiC Growth on Off-Oriented 4H-SiC Substrates. *Cryst. Growth Des.* **2015**, 15, 2940-2947. DOI: 10.1021/acs.cgd.5b00368.

(4) Jian, J.; Shi, Y.; Ekeroth, S.; Keraudy, J.; Syväjärvi, M.; Yakimova, R.; Helmersson, U.; Sun, J. A nanostructured NiO/cubic SiC p-n heterojunction photoanode for enhanced solar water splitting. *J. Mater. Chem. A* **2019**, 7, 4721-4728. DOI: 10.1039/C9TA00020H.

(5) Lauermann, I.; Memming, R.; Meissner, D. Electrochemical Properties of Silicon Carbide. *J. electrochem. Soc.* **1997**, 144, 73. DOI: 10.1149/1.1837367.

(6) Tae Song, J.; Mashiko, H.; Kamiya, M.; Nakamine, Y.; Ohtomo, A.; Iwasaki, T.; Hatano, M. Improved visible light driven photoelectrochemical properties of 3C-SiC semiconductor with Pt nanoparticles for hydrogen generation. *Appl. Phys. Lett.* **2013**, 103, 213901. DOI: 10.1063/1.4832333.

(7) Song, J. T.; Iwasaki, T.; Hatano, M. Pt co-catalyst effect on photoelectrochemical

properties of 3C-SiC photo-anode. *Jpn. J. Appl. Phys.* **2014**, 53, 05FZ04. DOI: 10.7567/JJAP.53.05FZ04.

(8) Sun, J. W.; Jokubavicius, V.; Gao, L.; Booker, I.; Jansson, M.; Liu, X. Y.; Hofmann, J. P.; Hensen, E. J.; Linnarsson, M. K.; Wellmann, P. J. Solar driven energy conversion applications based on 3C-SiC. *Materials Science Forum*, **2016**; 858, 1028-1031.

(9) Jian, J.; Shi, Y.; Syväjärvi, M.; Yakimova, R.; Sun, J. Cubic SiC photoanode coupling with Ni: FeOOH oxygen-evolution cocatalyst for sustainable photoelectrochemical water oxidation. *Solar RRL* **2020**, 4, 1900364. DOI: 10.1002/solr.201900364

(10) Li, H.; Shi, Y.; Shang, H.; Wang, W.; Lu, J.; Zakharov, A. A.; Hultman, L.; Uhrberg, R. I.; Syväjärvi, M.; Yakimova, R. Atomic-scale tuning of graphene/cubic SiC Schottky junction for stable low-bias photoelectrochemical solar-to-fuel conversion. *ACS nano* **2020**, 14, 4905-4915. DOI: 10.1021/acsnano.0c00986.

(11) Jian, J.-X.; Jokubavicius, V.; Syväjärvi, M.; Yakimova, R.; Sun, J. Nanoporous Cubic Silicon Carbide Photoanodes for Enhanced Solar Water Splitting. *ACS Nano* **2021**, 15, 5502-5512. DOI: 10.1021/acsnano.1c00256.
